# CSPG4-dependent cytotoxicity for *C. difficile* TcdB is influenced by extracellular calcium and chondroitin sulfate

**DOI:** 10.1128/msphere.00094-24

**Published:** 2024-03-12

**Authors:** D. Annie Doyle, Paul L. DeAngelis, Jimmy D. Ballard

**Affiliations:** 1 Department of Microbiology and Immunology, The University of Oklahoma Health Sciences Center, Oklahoma City, Oklahoma, USA; 2 Department of Biochemistry and Physiology, The University of Oklahoma Health Sciences Center, Oklahoma City, Oklahoma, USA; University of Kentucky College of Medicine, Lexington, Kentucky, USA

**Keywords:** *Clostridioides difficile*, TcdB, calcium, chondroitin sulfate

## Abstract

TcdB is an intracellular bacterial toxin indispensable to *Clostridioides difficile* infections. The ability to use chondroitin sulfate proteoglycan 4 (CSPG4) as a primary cell surface receptor is evolutionarily conserved by the two major variants of TcdB. As CSPG4 does not typically undergo receptor-mediated endocytosis, we sought to identify environmental factors that stabilize interactions between TcdB and CSPG4 to promote cell binding and entry into the cytosol. Using a series of TcdB receptor-binding mutants and cell lines with various receptor expression profiles, we discovered that extracellular Ca^2+^ promotes receptor-specific interactions with TcdB. Specifically, TcdB exhibits preferential binding to CSPG4 in the presence of Ca^2+^, with the absence of Ca^2+^ resulting in CSPG4-independent cell surface interactions. Furthermore, Ca^2+^ did not enhance TcdB binding to chondroitin sulfate (CS), the sole glycosaminoglycan of CSPG4. Instead, CS was found to impact the rate of cell entry by TcdB. Collectively, results from this study indicate that Ca^2+^ enhances cell binding by TcdB and CS interactions contribute to subsequent steps in cell entry.

**IMPORTANCE:**

*Clostridioides difficile* is a leading cause of antibiotic-associated gastrointestinal illness, and many disease pathologies are caused by the toxin TcdB. TcdB engages multiple cell surface receptors, with receptor tropisms differing among the variants of the toxin. Chondroitin sulfate proteoglycan 4 (CSPG4) is a critical receptor for multiple forms of TcdB, and insights into TcdB–CSPG4 interactions are applicable to many disease-causing strains of *C. difficile*. CSPG4 is modified by chondroitin sulfate (CS) and contains laminin-G repeats stabilized by Ca^2+^, yet the relative contributions of CS and Ca^2+^ to TcdB cytotoxicity have not been determined. This study demonstrates distinct roles in TcdB cell binding and cell entry for Ca^2+^ and CS, respectively. These effects are specific to CSPG4 and contribute to the activities of a prominent isoform of TcdB that utilizes this receptor. These findings advance an understanding of factors contributing to TcdB’s mechanism of action and contribution to *C. difficile* disease.

## INTRODUCTION

*Clostridioides difficile* TcdB is a 2,366 amino acid protein broadly classified as an A-B toxin in the family of large clostridial toxins (1). Like many other intracellular bacterial toxins, TcdB localizes to target cells using cell surface proteins as receptors to initiate toxin uptake (2, 3). During cell entry, TcdB undergoes autoprocessing, releasing its glucosyltransferase domain to subsequently hydrolyze UDP-glucose and transfer the liberated sugar moiety to a reactive threonine in the effector binding loop of the small GTPases Rho, Rac, and Cdc42 (4–6). These actions result in the manifestation of colonic inflammation, gross tissue damage, and impaired wound healing during disease (7–10). Without TcdB, *C. difficile* is avirulent, and critical disease pathologies are absent (11).

Though TcdB’s activities are indispensable to *C. difficile* disease, there is variability in the sequences of *tcdB* across different clades, most notably, between the two TcdB types commonly associated with the disease: *Cd630* (TcdB1) and *Cd027* (TcdB2) (7, 11–13). Studies have found that this sequence variability is responsible for differences in TcdB cell tropism and toxicity (13, 14). Moreover, recent studies show that this variation in cell tropism is associated with differences in TcdB receptor-binding repertoires. Specifically, TcdB1 predominantly binds three frizzled protein homologs (FZD1: *K*_D_ = 32 nM; FZD2: *K*_D_ = 19 nM; and FZD7: *K*_D_ = 27 nM) (15), chondroitin sulfate proteoglycan 4 (CSPG4) (*K*_D_ = 15.2 nM) (16), and nectin-3 (*K*_D_ = 53 ± 7 nM) (10). Due to key amino acid differences from TcdB1, TcdB2 is unable to utilize FZD proteins as receptors. Instead, TcdB2 can bind CSPG4 at its laminin G repeats (*K*_D_ = 5.4 nM) (16) and tissue factor pathway inhibitor (TFPI) in its K2 domain (*K*_D_ = 0.4 µM) (17). The environmental factors that could drive receptor tropism for TcdB, causing the toxin to utilize CSPG4 rather than FZD, have not yet been described.

In the current study, we examined the role Ca^2+^ plays in differences between TcdB1 and TcdB2 cell interactions and cytotoxicity. Our rationale for investigating Ca^2+^ in the context of cell tropism and differences in TcdB1 and TcdB2 cytotoxicity was guided by two key observations. Metal ion cofactors, such as Ca^2+^, have been shown to promote interactions between proteins and glycosaminoglycans (18–20). Unlike CSPG4, FZD proteins do not possess glycosaminoglycan modifications or a known Ca^2+^-binding domain, which supports the notion that Ca^2+^ could impact CSPG4 but not FZD tropism, with a greater effect on TcdB2. Second, previous studies from our lab identified a cell-penetrating peptide derived from amino acids 1769–1787 in TcdB2 that are necessary for cell binding and uptake (21, 22). Interestingly, some cell-penetrating peptides are known to utilize glycosaminoglycans for cell entry (23, 24), which would also be enhanced in the presence of Ca^2+^. As such, we hypothesize that Ca^2+^ influences cell surface interactions with TcdB, and this ion could contribute to differences in TcdB1 and TcdB2 receptor tropism.

Using TcdB mutants with altered receptor-binding capabilities, we show that TcdB preferentially utilizes CSPG4 over FZD in the presence of extracellular Ca^2+^. Furthermore, using a combination of biochemical approaches and cell lines defective in CSPG4 expression, we found that Ca^2+^ is important for TcdB cell binding in a CSPG4-dependent manner. Moreover, we identified chondroitin sulfate, the sole glycosaminoglycan of CSPG4, as an important factor for TcdB cell entry. Altogether, this study demonstrates how extracellular Ca^2+^ influences critical cell interactions with TcdB to promote cellular intoxication.

## RESULTS

### Calcium influences cellular interactions with TcdB

**Fig 1:**
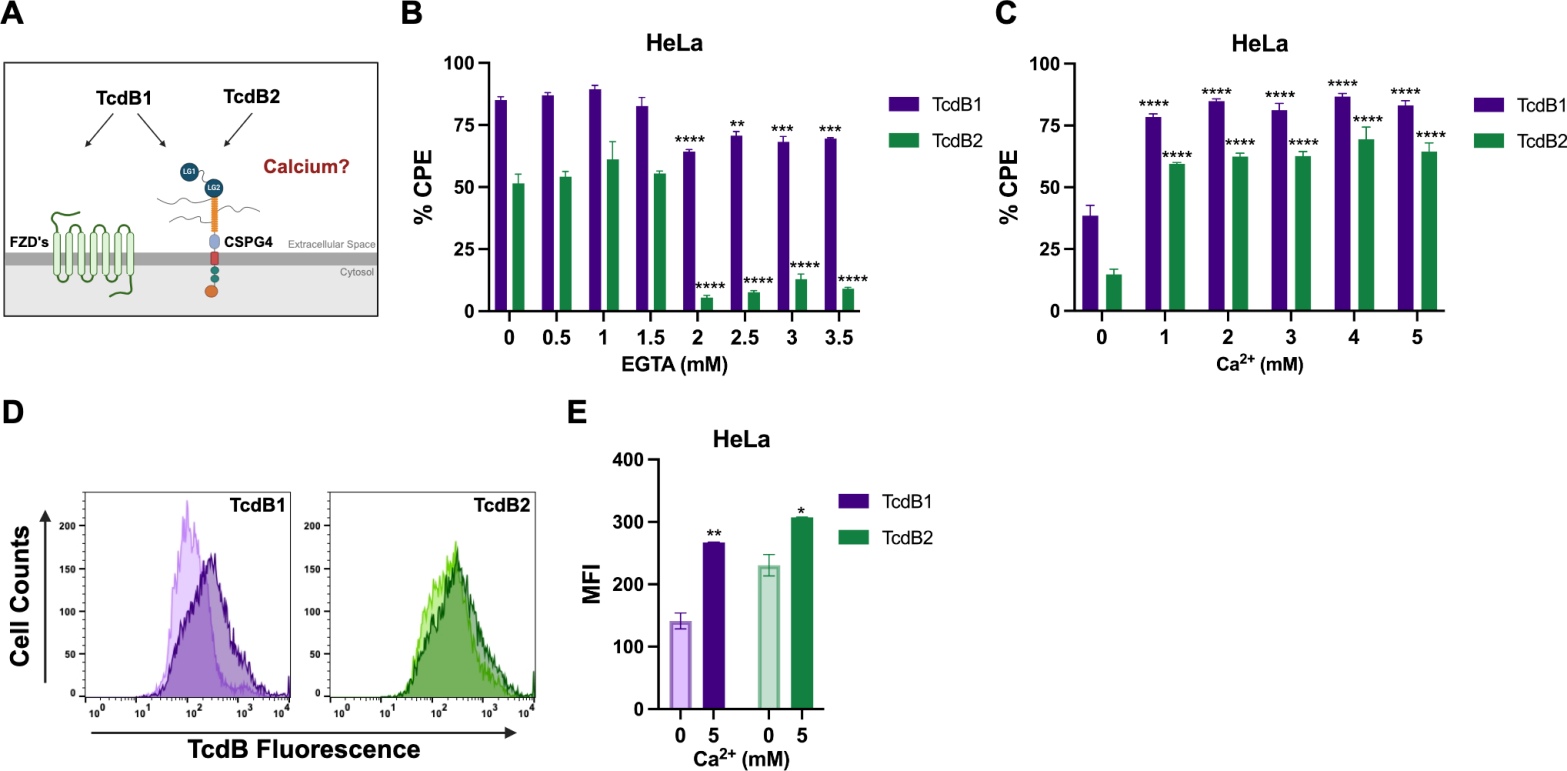
Divalent cations regulate TcdB cellular interactions and cytotoxicity. (**A**) Schematic showing the different receptor-binding capabilities of TcdB1 and TcdB2. Cytotoxicity assays were used to quantify the % CPE in HeLa cells (*n* = 3) following a 3-h treatment with 10 pM TcdB1 or TcdB2 when (**B**) HeLa cells were pretreated with EGTA. (**C**) TcdB was administered in HBSS containing 0–5 mM CaCl_2_. Results are given as the mean ± standard error of the mean from a representative experiment. Each experiment was repeated three independent times with similar results. (**D and E**) TcdB binding to HeLa cells was measured by flow cytometry. Cells were exposed to 30 nM TcdB1_AlexaFluor647_ or TcdB2_AlexaFluor647_ in HBSS ± 5 mM CaCl_2_ for 10 min at 37°C followed by 20 min on ice before a series of washes and assessment by flow cytometry. (**D**) Histograms comparing the fluorescent signaling from HeLa cells exposed to TcdB1_AlexaFluor647_ or TcdB2_AlexaFluor647_ in the presence (dark color shade) or absence (light color shade) of CaCl_2_. (**E**) Mean fluorescent intensity (MFI) of a representative flow cytometry experiment given as the mean ± standard error of the mean. Each experiment was repeated two independent times with similar results. Statistical significance for each experiment was calculated using a two-way ANOVA with Šídák’s multiple comparisons test. ^*^P ≤ 0.03; ^**^P ≤ 0.002; ^***^P ≤ 0.0002; ^****^P ≤ 0.0001.

Ca^2+^ is involved in multiple facets of *C. difficile* pathogenesis (25, 26), yet whether Ca^2+^ serves a functional role in the interactions between TcdB and target cells has yet to be determined (Fig. 1A). To examine this, HeLa cells were treated with 10 pM TcdB1 or TcdB2 following the chelation of extracellular Ca^2+^ using the membrane-impermeable form of EGTA. In the absence of free Ca^2+^, the cytopathic effects (CPEs) of TcdB2 but not TcdB1 were significantly reduced (Fig. 1B) regardless of the toxin concentration used or the length of toxin exposure (Fig. S1A and B). To measure the influence of Ca^2+^ on TcdB function in the absence of complex media, HeLa cells were treated with 10 pM TcdB1 or TcdB2 in divalent cation-free Hanks Balanced Salt Solution (HBSS) supplemented with 0–5 mM CaCl_2_. The CPE of TcdB1 and TcdB2 significantly increased in the presence of Ca^2+^ at 3 and 6 h post-treatment (Fig. 1C; Fig. S1C). In line with the enhanced cytotoxicity, HeLa cells treated with 30 nM TcdB1_AlexaFluor647_ or TcdB2_AlexaFluor647_ showed increased cell surface binding in the presence of Ca^2+^ when measured by flow cytometry (Fig. 1D and E). Furthermore, the level of TcdB detected from HeLa cell lysates treated with toxin in the presence of Ca^2+^ increased (Fig. S1D). These observations collectively show that the presence of Ca^2+^ enhances cell binding of both TcdB variants yet has a greater contribution to the CPE of TcdB2 compared to TcdB1. Given that these TcdB variants differ in receptor-binding strategies, we posited that these differences in the CPE between TcdB1 and TcdB2 are receptor-specific.

### Calcium promotes preferential binding to CSPG4 over FZD

**Fig 2:**
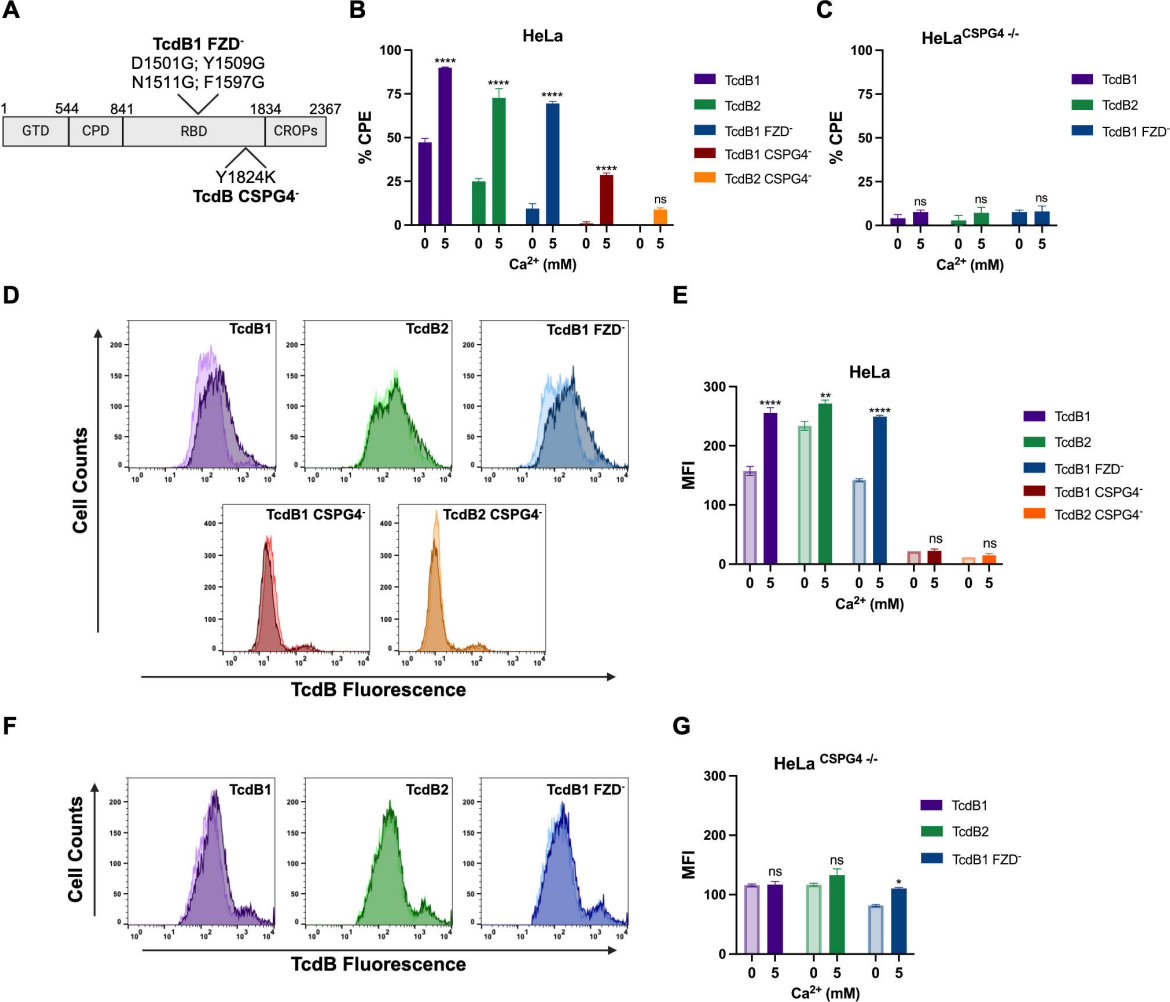
Calcium regulates interactions between TcdB and CSPG4. (**A**) Schematic of the amino acid substitutions in TcdB receptor-binding mutants. (**B**) Cytotoxicity assay used to quantify the % CPE in HeLa cells (*n* = 3) following a 3-h treatment with 10 pM TcdB1, TcdB2, TcdB1^FZD−^, TcdB1^CSPG4−^, or TcdB2^CSPG4−^ in HBSS ±5 mM CaCl_2_. (**C**) Cytotoxicity assay used to quantify the % CPE in HeLa^CSPG4−/−^ cells (*n* = 3) following a 3-h treatment with 10 pM TcdB1, TcdB2, or TcdB1^FZD−^ in HBSS ± 5 mM CaCl_2_. Results are given as the mean ± standard error of the mean from a representative experiment. Each experiment was repeated three independent times with similar results. (**D–G**) TcdB binding to HeLa cells (*n* = 3) or HeLa^CSPG4−/−^ cells (*n* = 3) was measured by flow cytometry. Cells were exposed to 30 nM TcdB1_AlexaFluor647_, TcdB2_AlexaFluor647_, TcdB1^FZD−^_AlexaFluor647_, TcdB1^CSPG4−^_AlexaFluor488_, or TcdB2^CSPG4−^_AlexaFluor488_ in HBSS ± 5 mM CaCl_2_ for 10 min at 37°C followed by 20 min on ice before a series of washes and assessment by flow cytometry. (**D and F**) Histograms comparing the fluorescent signal from HeLa cells and HeLa^CSPG4−/−^ cells exposed to TcdB1_AlexaFluor647_, TcdB2_AlexaFluor647_, TcdB1^FZD−^_AlexaFluor647_, TcdB1^CSPG4−^_AlexaFluor488_, or TcdB2^CSPG4−^_AlexaFluor488_ in the presence (dark color shade) or absence (light color shade) of CaCl_2_. (**E and G**) MFI of a representative flow cytometry experiment given as a mean ± standard error of the mean. Each experiment was repeated two independent times with similar results. Statistical significance for each experiment was calculated using a two-way ANOVA with Šídák’s multiple comparisons test. ^*^P ≤ 0.03; ^**^P ≤ 0.002; ^****^P ≤ 0.0001.

The ability to bind CSPG4 is evolutionarily conserved between TcdB1 and TcdB2. However, the divergence of TcdB2 from TcdB1 resulted in the loss of FZD binding (3). As shown in Fig. 1, TcdB1 induces a significant CPE in the absence of Ca^2+^, suggesting that TcdB1 does not require Ca^2+^ to utilize FZD for cell entry. Using a series of TcdB mutants lacking the ability to bind either FZD or CSPG4, we examined the effect of Ca^2+^ on TcdB cellular interactions (Fig. 2A). In the absence of Ca^2+^, HeLa cells treated with TcdB1^FZD−^ exhibited a significant reduction in CPE, with the presence of Ca^2+^ having increased the CPE and cell surface associations similar to that of wild-type (WT) TcdB (Fig. 2B, D, and E; Fig. S2 and S3). Furthermore, Ca^2+^ had no effect on the CPE or cell surface binding in HeLa^CSPG4−/−^ cells treated with TcdB (Fig. 2C, F, and G; Fig. S4). To examine the effects of Ca^2+^ when CSPG4 binding is diminished in TcdB, a Y1824K mutation was generated in TcdB1 and TcdB2 (Fig. 2A) as originally described by Gupta et al. (27). Compared to WT TcdB, treatment of HeLa cells with TcdB1^CSPG4−^ in the presence of Ca^2+^ resulted in a slight increase in CPE after 3 h (Fig. 2B; Fig. S2), with a substantial increase in the CPE of TcdB1^CSPG4−^ and TcdB2^CSPG4−^ occurring after 6 h (Fig. S3). Interestingly, Ca^2+^ did not increase cell surface binding for either TcdB1^CSPG4−^_AlexaFluor488_ or TcdB2^CSPG4−^_AlexaFluor488_ (Fig. 2D and E). TcdB1 and TcdB2 interact with CSPG4 using multiple contact residues, which have been shown to differ between the two variants (16, 27). We suspect that in our experimental system, these TcdB Y1824K mutants do not completely diminish CSPG4 binding over an extended incubation period but, instead, function as lower affinity CSPG4-binding mutants. Nevertheless, these data suggest that TcdB preferentially utilizes CSPG4 over FZD as its primary surface receptor in a Ca^2+^-dependent manner.

### Calcium-enhanced TcdB binding is independent of glycosaminoglycans

**Fig 3:**
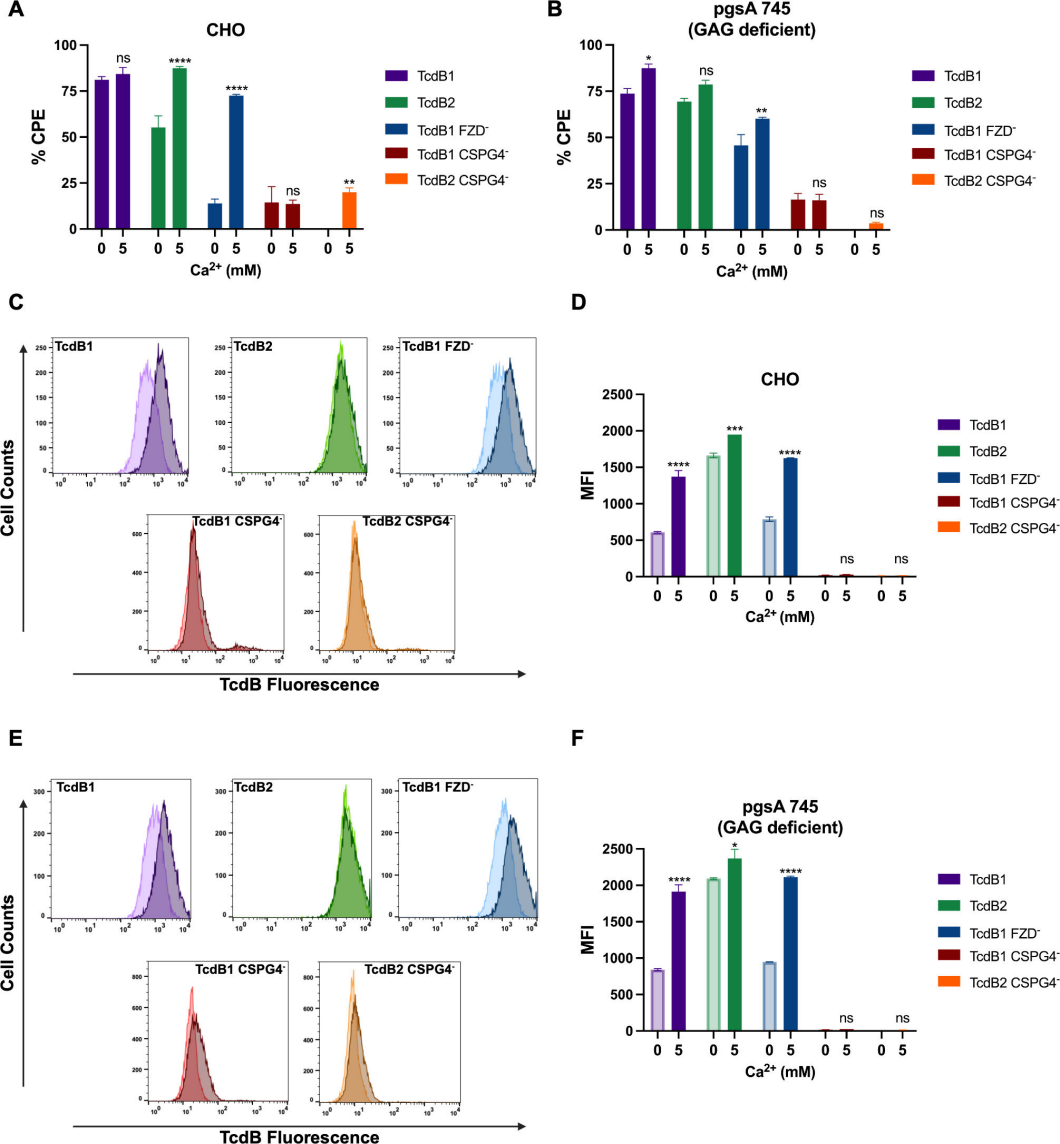
Calcium-enhanced cell surface binding is independent of glycosaminoglycans. (**A and B**) Cytotoxicity assay used to quantify the % CPE in wild-type CHO cells (*n* = 3) and CHO pgsA745 cells (*n* = 3) following a 2-h treatment with 10 pM TcdB1, TcdB2, TcdB1^FZD−^, TcdB1^CSPG4−^, or TcdB2^CSPG4−^ in HBSS ± 5 mM CaCl_2_. Results are given as the mean ± standard error of the mean from a representative experiment. Each experiment was repeated three independent times with similar results. (**C–F**) TcdB binding to CHO cells or CHO pgsA745 cells was measured by flow cytometry. Cells were exposed to 30 nM TcdB1_AlexaFluor647_, TcdB2_AlexaFluor647_, or TcdB1^FZD−^_AlexaFluor647_ in HBSS ± 5 mM CaCl_2_ for 10 min at 37°C followed by 20 min on ice before a series of washes and assessment by flow cytometry. (**C and E**) Histograms comparing the fluorescent signal from CHO cells and CHO pgsA745 cells exposed to TcdB1_AlexaFluor647_, TcdB2_AlexaFluor647_, TcdB1^FZD−^_AlexaFluor647_, TcdB1^CSPG4−^_AlexaFluor488_, or TcdB2^CSPG4−^_AlexaFluor488_ in the presence (dark color shade) or absence (light color shade) of 5 mM CaCl_2_. (**D and F**) MFI of flow cytometry data from a representative experiment. Results are given as the mean ± standard error of the mean. Each experiment was repeated two independent times with similar results. Statistical significance for each experiment was calculated using a two-way ANOVA with Šídák’s multiple comparisons test. ^*^P ≤ 0.03; ^**^P ≤ 0.002; ^****^P ≤ 0.0001.

CSPG4 is a large type 1 transmembrane protein composed of three distinct extracellular subdomains (D1–D3) (28). Ca^2+^ has been shown to independently promote protein interactions with both laminin G repeats (D1) and glycosaminoglycan chains (D2) (18, 29). As such, to investigate whether the Ca^2+^-dependent effects between TcdB and CSPG4 are specific to the laminin G repeats or the glycosaminoglycan chains of CSPG4, we utilized the CHO pgsA-745 cell line. This cell line produces the core CSPG4 protein but lacks the formation of the tetrasaccharide core junction between the CS chains and the protein due to mutagenesis of the xylosyltransferase gene (30). While the presence of Ca^2+^ did not have as drastic of an impact on the CPE of TcdB in CHO pgsA-745 cells compared to WT CHO cells (Fig. 3A and B), Ca^2+^ significantly enhanced the cell binding of TcdB1_AlexFluor647_ and TcdB1^FZD−^_AlexaFluor647_ in both WT CHO cells and CHO pgsA-745 cells. Interestingly, Ca^2+^ only marginally increased TcdB2_AlexaFluor647_ cell surface binding in WT CHO cells (Fig. 3C through F). Furthermore, the addition of Ca^2+^ had no effect on the CPE nor the overall cell binding of TcdB1^CSPG4−^ or TcdB2^CSPG4−^ to either WT CHO or CHO pgsA-745 cells (Fig. 3A through F). These data suggest that Ca^2+^ most likely promotes interactions with the laminin G repeats of CSPG4, but additional interactions between TcdB and CSPG4 could impact the overall cytotoxicity of TcdB.

### Interactions with chondroitin sulfate influence TcdB cytotoxicity

**Fig 4:**
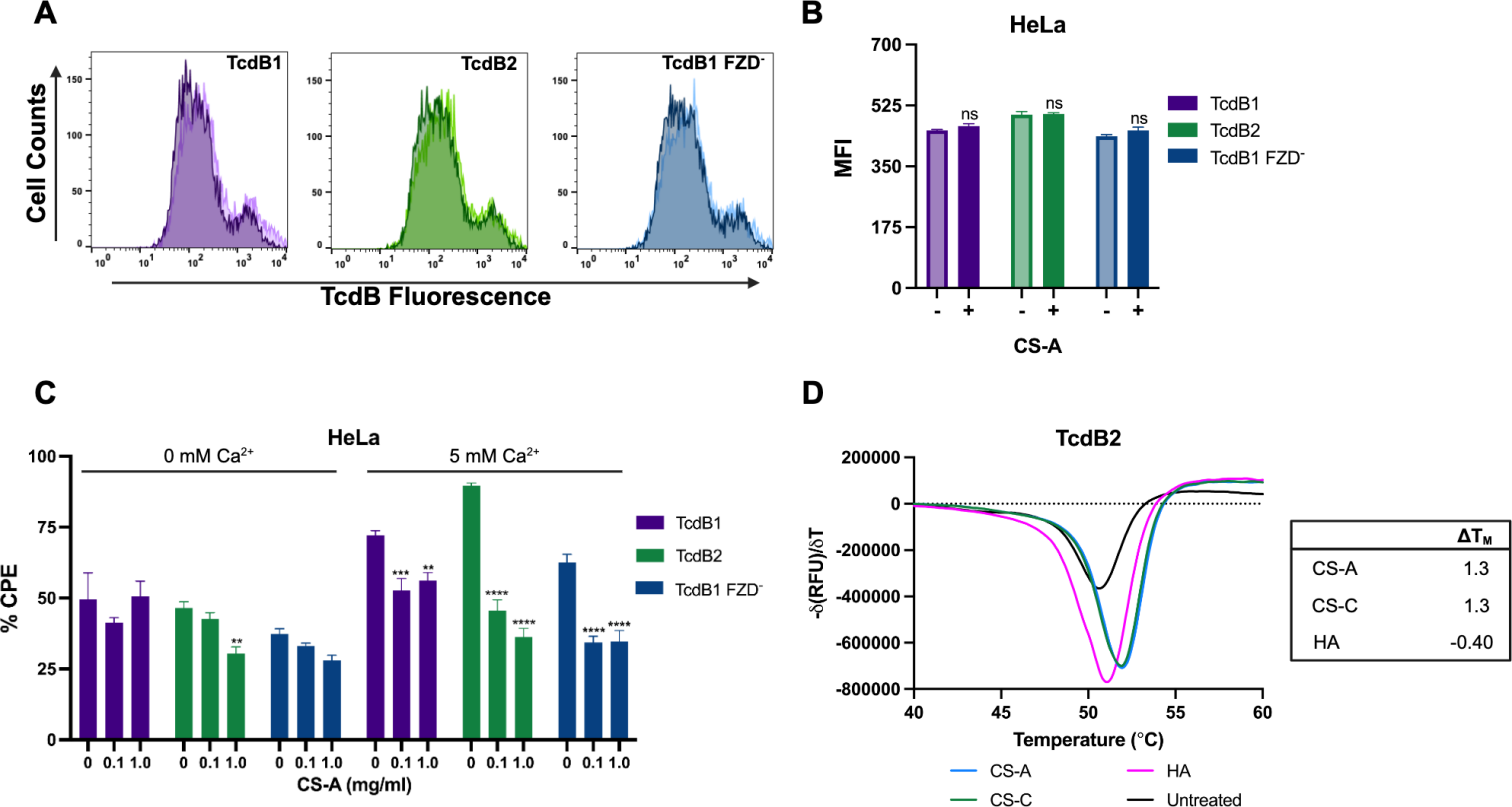
Interactions with chondroitin sulfate regulate TcdB cytotoxicity. (**A and B**) TcdB binding to HeLa cells in the presence of soluble chondroitin sulfate was measured by flow cytometry. Cells were exposed to a mixture of 30 nM TcdB1_AlexaFluor647_, TcdB2_AlexaFluor647_, or TcdB1^FZD−^_AlexaFluor647_ with 8.0 mg/mL CS-A in HBSS + 5 mM CaCl_2_ for 10 min at 37°C followed by 20 min on ice before a series of washes and assessment by flow cytometry. (**A**) Histograms comparing the fluorescent signal from HeLa cells exposed to TcdB1_AlexaFluor647_, TcdB2_AlexaFluor647_, or TcdB1^FZD−^_AlexaFluor647_ in the presence (dark color shade) or absence (light color shade) of chondroitin sulfate. (**B**) MFI of flow cytometry data from a representative experiment. Results are given as the mean ± standard error of the mean from a representative experiment given as a mean ± standard error of the mean. Each experiment was repeated two independent times with similar results. Cytotoxicity assays were used to quantify the % CPEs in HeLa cells (*n* = 3) following a 2-h treatment of 10 pM TcdB1, TcdB2, or TcdB1^FZD−^ mixed with soluble CS-A (**C**) in the presence or absence of 5 mM CaCl_2_. Differential scanning fluorimetry was used to measure changes in the thermal stability of TcdB2 when 3-µg TcdB2 is incubated with (**D**) 200 µM CS-A, 200 µM CS-C, or 100 µM HA in HBSS + 5 mM CaCl_2_. The inverse first derivative with Δ*T*_m_ values is provided for each TcdB thermal denaturation run. Results are given as the mean ± standard error of the mean from a representative experiment. Each experiment was repeated three independent times with similar results. Statistical significance for each experiment was calculated using a two-way ANOVA with Šídák’s multiple comparisons test. ^*^P ≤ 0.03; ^**^P ≤ 0.002; ^***^P ≤ 0.0002; ^****^P ≤ 0.0001.

Cell surface proteoglycans possess covalently attached sulfated glycosaminoglycan (sGAG) polymers that interact with nearby proteins to regulate various cell signaling events, including endocytosis (31, 32). These interactions are largely electrostatic, driven by the frequency and location of negatively charged sugar-linked sulfate groups. Additionally, metal cofactors, such as Ca^2+^, can further facilitate interactions between proteins and sGAGs, such as chondroitin sulfate (CS) (18, 19). As the GAG chains of CSPG4 consist solely of CS, we asked whether the Ca^2+^-dependent interactions between TcdB and CSPG4 involved CS. To determine whether interactions with soluble CS influenced TcdB cytotoxicity, TcdB was premixed with two different forms of soluble CS resembling the chains attached to CSPG4. Cells treated with TcdB1, TcdB2, or TcdB1^FZD−^ mixed with CS-A in the presence of Ca^2+^ had a significant reduction in CPE after 2 and 4 h (Fig. 4C; Fig. S5D). Comparatively, HeLa cells treated with CS-C premixed with TcdB1 or TcdB2, but not TcdB1^FZD−^, had a slight reduction in CPE after 2 h but not 4 h (Fig. S5C and E). Interestingly, neither CS-A nor CS-C had a substantial impact on TcdB cell surface binding when HeLa cells were treated with 30 nM TcdB1_AlexaFluor647_, TcdB2_AlexaFluor647_, or TcdB1^FZD−^_AlexaFluor647_ and examined via flow cytometry (Fig. 4A and B; Fig. S5A and B). As the addition of soluble CS did not block toxin binding but did mitigate TcdB2 cytotoxicity, we examined whether stabilizing interactions between TcdB and soluble CS could be detected using differential scanning fluorimetry (DSF). While CS-A had a greater effect on the CPE of TcdB2 than CS-C, the stabilization of TcdB2 was not dependent on whether the *N*-acetylgalactosamine residues of CS were predominantly sulfated at position C-4 (CS-A Δ*T*_M_ = 1.3°C) or C-6 (CS-C Δ*T*_M_ = 1.3°C) but did show a dependency on the presence of negatively charged sulfate groups (HA Δ*T*_M_ = −0.40°C) (Fig. 4D). While the addition of CS resulted in a relatively low shift in melting temperature, there was a significant effect of CS on the CPE of TcdB. A similar profile was observed by Tam et al. in which the CPE of TcdB was significantly impacted by the addition of various bile salts but had a relatively low stabilizing effect on TcdB when examined via DSF (33).

### The rate of TcdB cell entry is impacted by interactions with chondroitin sulfate

**Fig 5:**
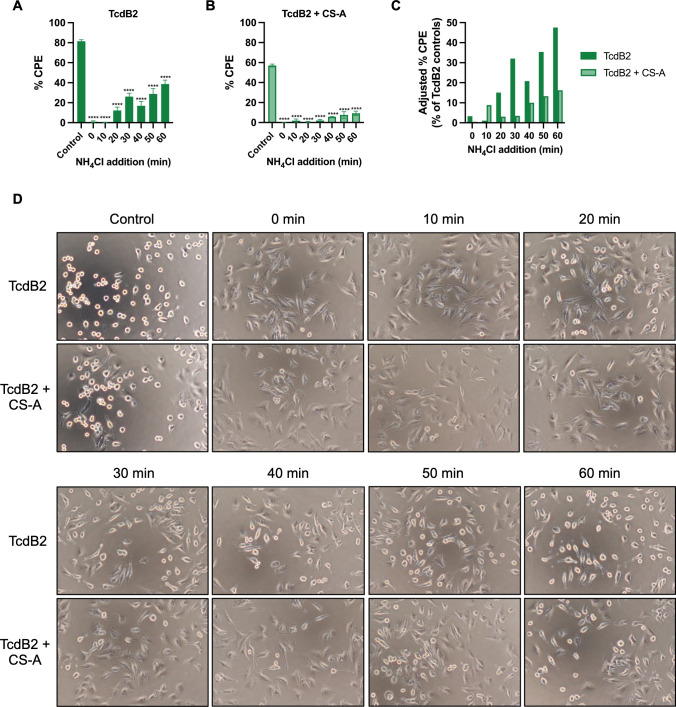
Chondroitin sulfate influences the rate of TcdB cell entry. Lysosomotropic inhibitor assays used to determine the rate of toxin cell entry based on changes in % CPE between treatment groups. HeLa cells (*n* = 3) were treated with (**A**) 10 pM TcdB2 or (**B**) 10 pM TcdB2 + 1 mg/mL CS-A in HBSS + 5 mM CaCl_2_ with NH_4_Cl added to each well for a final concentration of 25 mM at the indicated time points. Cytopathic effects were determined after 3 h. (**C**) Normalization of the % CPE from TcdB2 and TcdB2 + CS-A groups treated with NH_4_Cl. (**D**) Representative microscopy images of HeLa cells at 3 h. Cells intoxicated by TcdB can be distinguished from non-intoxicated cells if the cell is >95% rounded in appearance due to cytoskeletal collapse. Results are given as the mean ± standard error of the mean from a representative experiment. Each experiment was repeated three independent times with similar results. Statistical significance for each experiment was calculated using a one-way ANOVA with Dunnett’s multiple comparisons test. ^****^P < 0.0001.

The efficiency of TcdB endocytosis has been shown to vary between the TcdB1 and TcdB2 variants. We have found that interactions with CS influence TcdB in the presence of Ca^2+^, yet whether this is a result of impaired cell entry remained unknown. To examine whether interactions with CS impact the rate at which TcdB2 enters cells, HeLa cells were treated with or without 10 pM TcdB2 ± 1 mg/mL CS-A in HBSS + 5 mM CaCl_2_. The lysosomotropic inhibitor NH_4_Cl, which disrupts charge gradients across the endosomal membrane (34), was added in 10-min increments for a total of 1 h, starting at time 0. While NH_4_Cl reduced the rate of cell rounding by TcdB2 alone after 3 h (Fig. 5A through D), the presence of CS-A further delayed the CPE of TcdB2 (Fig. 5B through D). This observation indicates that interactions between TcdB2 and CS are directly associated with alterations in the rate of toxin uptake. As such, these data suggest a mechanism for TcdB cell entry that is independent of initial cell surface interactions but is dependent on Ca^2+^-driven interactions with the glycosaminoglycan chains of CSPG4.

## DISCUSSION

TcdB1 and TcdB2 are the most frequent toxin variants identified in the clinic from *C. difficile*-infected patients (12, 35, 36). These TcdB variants share 92% sequence similarity, contributing to differences in receptor tropism and pathological severity (13, 14). It remains unclear why TcdB2 evolved to lose FZD binding and preferentially utilize CSPG4 but could be a potential means to target certain tissues or compartments of the gastrointestinal tract. Furthermore, the mechanism by which TcdB triggers cell entry following receptor binding appears to differ between TcdB1 and TcdB2.

Both commensal and pathogenic microorganisms take advantage of specific environmental factors to enhance their fitness, as is the case with *C. difficile* and Ca^2+^. Exogenous Ca^2+^ works in tandem with bile salts as a cogerminant of *C. difficile* spores, leading to colonization by vegetative cells and the development of disease (26). Additionally, high concentrations of TcdB induce an influx of extracellular Ca^2+^ required for cell necrosis, contributing to *C. difficile*’s nutritional niche (25). Yet, whether Ca^2+^ influences the cellular interactions of TcdB and its receptor tropism had not been investigated. In support of our initial prediction, the presence of extracellular Ca^2+^ increased TcdB binding to host cells, regardless of the TcdB variant used. However, significant differences in cytotoxicity were observed between TcdB1 and TcdB2 in the absence of extracellular Ca^2+^ (Fig. 1). We reasoned that these differences in cytotoxicity were receptor-specific, with TcdB intoxication through CSPG4 dependence on the presence of extracellular Ca^2+^.

The data shown in Fig. 2 suggest that the effect of Ca^2+^ on TcdB receptor binding is most relevant to CSPG4. As previously noted, TcdB2 has a higher affinity for CSPG4 compared to TFPI, with TcdB1 possessing a similar affinity for both FZD and CSPG4 (15–17). In the absence of Ca^2+^, cytotoxicity was significantly reduced following treatments with either TcdB1^FZD−^ or TcdB2 (Fig. 2). Yet, the presence or absence of Ca^2+^ was of minimal consequence when cells were treated with TcdB1. These data suggest that Ca^2+^ availability could influence TcdB1’s ability to utilize FZD and CSPG4 as receptors under different environmental conditions. In line with this, TcdB1^CSPG4−^ and TcdB2^CSPG4−^ showed a substantial reduction in the CPE and cell surface association on HeLa cells in both the presence and absence of Ca^2+^. Moreover, compared to WT HeLa cells, Ca^2+^ had no impact on the CPE or cell surface associations in HeLa^CSPG4−/−^ cells when treated with either TcdB1 or TcdB2, further suggesting CSPG4 functions as the preferential cell surface receptor for these two TcdB variants on these cells (Fig. 2). This selectivity for CSPG4 corresponds with recent findings by Childress et al., which found TcdB1 preferentially localized with CSPG4 over nectin-3 (37). Furthermore, our data suggest that Ca^2+^ enhances specific interactions between TcdB and CSPG4 to promote cell surface binding and intoxication.

CSPG4 is a type 1 transmembrane protein that does not typically undergo receptor-mediated endocytosis but rather is cleaved from the cell surface to function as a potent signaling molecule (38–42). To effectively utilize CSPG4 during cellular intoxication, TcdB interactions with CSPG4 must be stabilized to prevent receptor shedding and allow for the toxin to be endocytosed. Our data suggest that Ca^2+^ could contribute to this stabilizing interaction between TcdB and CSPG4. Indeed, previous studies have shown that Ca^2+^ promotes interactions between proteins and laminin G repeat domains (29), as well as between proteins and GAGs (18, 19). The effects of Ca^2+^ on TcdB binding and cytotoxicity align with these previous findings. Our study shows that Ca^2+^ had a greater impact on TcdB cell surface binding than overall TcdB cytotoxicity in the GAG-deficient CHO pgsA-745 cell line (Fig. 3), thus suggesting that Ca^2+^ could enhance TcdB binding to the laminin G repeats of CSPG4 rather than CS. The structure of TcdB1 binding to a laminin G repeat of CSPG4 was published by Chen et al. in 2021 (16). Although the cryo-EM structure did not include Ca^2+^, we note that aspartic acid residues in the laminin repeat (D457 and D498) were found in the contact regions, and mutagenesis studies showed that D1812 in the hinge region of TcdB was required for cell association and cytotoxicity. Aspartic acids are essential for Ca^2+^ binding but require coordination with other proximal amino acids, and these were not apparent in the high-resolution structure. Thus, we suspect that the more likely mechanism involves Ca^2+^ stabilization of the laminin G repeats in CPSG4.

While Ca^2+^ was shown to enhance toxin binding in the absence of GAGs, we wondered if more specific interactions with the CS chains of CSPG4 could influence TcdB cytotoxicity and cell entry. Using DSF, we identified sulfation-dependent, stabilizing interactions between soluble CS and TcdB in the presence of Ca^2+^ (Fig. 4). The Δ*T*_M_ found in this analysis were not dramatic, suggesting a low-affinity interaction between TcdB and CS. Yet, this is not unexpected, as it is not uncommon for proteins that utilize sGAGs as cofactors to bind at low affinities. For example, the HS chains of HS proteoglycans function as a coreceptor for FGF-2 by decreasing the concentration of FGF-2 needed for FGFR activation. Specifically, FGF-2 binding HS localizes the growth factor to the vicinity of the cell surface (43, 44). Our study shows that in the presence of Ca^2+^, interactions with CS significantly affect the cytotoxicity of TcdB without interfering with cell surface binding, leading us to suspect this could be involved in triggering cell entry following binding to CSPG4. Moreover, it is possible that Ca^2+^ drives binding to the laminin G repeats and thus enhancing TcdB interactions with the CS chains of CSPG4 to immobilize the TcdB:CSPG4 complex on the plasma membrane for internalization.

Considering CS caused a significant reduction in TcdB cytotoxicity without impacting cell binding or causing a dramatic change in TcdB’s Δ*T*_M_, we wondered if CS was impacting the efficiency of cell entry rather than initial steps in receptor binding. Using the lysosomotropic inhibitor NH_4_Cl, we monitored the rate of TcdB cell entry in the presence and absence of CS-A. As shown in Fig. 5, CS-A significantly delayed TcdB2 cell entry, suggesting that Ca^2+^-driven interactions with CS play a role in the rate of TcdB uptake. Competitive inhibition of CS binding necessary for triggering cell entry could explain why we detected no difference in cell binding but a significant reduction in cytotoxicity. Furthermore, earlier studies found that TcdB2 entered cells more rapidly than TcdB1 (14, 45), so it is plausible that this behavior is due to TcdB2’s interactions with CS following binding to protein regions of CSPG4.

TcdA, the second large clostridial toxin produced by *C. difficile*, is related to TcdB but differs in its receptor-binding profiles. Tao et al. reported that TcdA utilizes low-density lipoprotein and sulfated GAGs to engage cells (46). Differing from our findings with TcdB, sulfated GAGs were critical for TcdA binding to cells and not limited to mediating steps in cell entry. Like what was reported for TcdA, in our study, the sulfation of GAGs was important for interactions with TcdB. Thus, the sulfation of GAGs that likely impacts charge interactions is important to the cytotoxicity of both forms of the toxin.

CSPG4 is the largest and most structurally complex protein found on the surface of human cells (38). Its size, glycosylation, and flexibility have hindered efforts in the purification of full-length CSPG4, resulting in significant limitations and challenges when studying CSPG4–protein interactions. Despite this obstacle, we identified several Ca^2+^-dependent, CSPG4-specific interactions with the *C. difficile* toxin TcdB. Ongoing studies examining how TcdB interactions disrupt CSPG4 signaling pathways to promote toxin uptake will lead to a better understanding of toxin–receptor interactions.

## MATERIALS AND METHODS

### Cell lines and reagents

The human cervical epithelial cell line HeLa was purchased from the American Type Culture Collection. The HeLa Cas-9*^CSPG4−^*^/−^ cell line was a gift from Dr. Min Dong at Harvard Medical School generated as described in Tao et al. (15). HeLa and HeLa*^CSPG4−^*^/−^ cells were cultured in Eagles minimum essential medium (EMEM) supplemented with 10% fetal bovine serum (FBS), 100 units/mL penicillin, and 100 µg/mL streptomycin. All cell lines were grown at 37°C in the presence of 5% CO_2_.

### Construction and purification of TcdB mutants

Recombinant TcdB1 and TcdB2 were expressed in a *Bacillus megaterium* system (MoBiTec, Göttingen, Germany) as previously described and affinity-purified with Ni^2+^ chromatography (47). The TcdB1^FZD−^ mutant was expressed and purified as previously described (48). Plasmids encoding Y1824K in *tcdB1* (pC-His1522-tcdB1) and *tcdB2* (pC-His1522-tcdB2) were synthesized by GenScript, transformed into NEB 10-β *Escherichia coli* (New England Biolabs), and further expressed and purified in the *B. megaterium* system described above. Confirmation of appropriate mutations and the absence of off-target mutations were confirmed by DNA sequencing.

### Cytopathic cell-rounding assays

The respective cell lines were seeded into a 96-well plate at a density of 1 × 10^4^ per well and incubated at 37°C overnight to allow cell adherence. Cells were washed with either EMEM or F12K medium and then treated with toxin in calcium- and magnesium-free HBSS (Gibco cat no. 14175-095) with or without the addition of 5 mM CaCl_2_ and/or 5 mM MgCl_2_. Cells were imaged using an Olympus IX51 inverted microscope (Olympus, Waltham, MA), and the cytopathic effect (%CPE) was calculated as % rounded cells_(test)_ − % rounded cells_(control)_.

### Fluorescent protein labeling

Primary amines of TcdB were fluorescently labeled using the Alexa Fluor 647 and Alexa Fluor 488 protein labeling kits from ThermoFisher (catalog numbers A20173 and 10235) following the manufacturer’s protocol. The extinction coefficient of TcdB was predicted using the ExPASy ProtParam tool, and the degree of labeling for TcdB1, TcdB2, TcdB1^FZD−^, TcdB1^CSPG4−^, and TcdB2^CSPG4−^ was approximately 10 mol of dye per mol of protein.

### Flow cytometry

Cells were exposed to 30 nM TcdB1_AlexaFluor647_, TcdB2_AlexaFluor647_, TcdB1^FZD−^_AlexaFluor647_, TcdB1^CSPG4−^_AlexaFluor488_, and TcdB2^CSPG4−^_AlexaFluor488_ in HBSS (Gibco cat no. 14175-095) ± 5 mM CaCl_2_ for 10 min at 37°C followed by 20 min on ice before a series of washes and assessment by flow cytometry. Cell-associated fluorescence was quantified using a FACSCalibur flow cytometer or Stratedigm S1200Ex (University of Oklahoma Health Sciences Center). All data were analyzed using FlowJo software version 10.7.2 (Tree Star, Inc., San Carlos, CA).

### TcdB cell association assay

HeLa cells were seeded at 5 × 10^5^ in 12-well plates and incubated overnight at 37°C. The following day, cells were washed with EMEM and then treated with or without 200 pM TcdB1 or TcdB2 in HBSS (Gibco cat no. 14175-095) ± 5 mM CaCl_2_ and incubated at 37°C for 30 min. Cells were washed with HBSS ± 5 mM CaCl_2_, lysed with 250 µL lysis buffer [1% SDS, 50 mM Tris-HCl, 5 mM EDTA, pH 8, 1× Halt Protease Inhibitor Cocktail, EDTA-Free (ThermoFisher #87785)], and placed on ice for 15 min. Fifteen micrograms of total lysate was resolved by SDS-PAGE (8%) and transferred to a PVDF membrane. The membrane was probed with antibodies to the amino-terminal domain of TcdB at 1:200 (R&D Systems #AF6246). The immunoblots were developed using the Clarity Western ECL substrate (Bio-Rad #1705061) and imaged on a Bio-Rad ChemiDoc MP system.

### Chondroitin sulfate competition assay

Cytotoxicity assays were used to quantify the % CPE in HeLa cells following a 2- or 4-h treatment of 10 pM TcdB1, TcdB2, or TcdB1^FZD−^ mixed with 0.1 or 1.0 mg/mL soluble chondroitin sulfate A (Bovine; Bioiberica #F042510, Spain) or chondroitin sulfate C (Shark; Bioiberica #F042610, Spain) in HBSS ± 5 mM CaCl_2_. TcdB binding to HeLa cells in the presence of soluble chondroitin sulfate was measured by flow cytometry. HeLa cells were exposed to 30 nM TcdB1_AlexaFluor647_, TcdB2_AlexaFluor647_, or TcdB1^FZD−^_AlexaFluor647_ mixed with 8.0 mg/mL chondroitin sulfate A or chondroitin sulfate C in HBSS + 5 mM CaCl_2_ for 10 min at 37°C followed by 20 min on ice before a series of washes and assessment by flow cytometry.

### Lysosomotropic inhibitor assay

HeLa cells were plated at 1 × 10^4^ per well and incubated at 37°C overnight. The following day, cells were washed with fresh EMEM and then treated with 10 pM TcdB2 with or without 1 mg/mL CS-A in HBSS (Gibco cat no. 14175-095) containing 5 mM CaCl_2_. NH_4_Cl (Sigma #A4514) in HBSS containing 5 mM CaCl_2_ was added to each well for a final concentration of 25 mM in 10-min increments for a total of 1 h. Cells were imaged after 3 h of the addition of TcdB2 using an Olympus IX51 inverted microscope (Olympus, Waltham, MA), and the % CPE was calculated as described above with the control group being HBSS containing 5 mM CaCl_2_ with or without NH_4_Cl. The adjusted % CPE was calculated by subtracting the % CPE of TcdB2 or TcdB2 + CS-A NH_4_Cl-treated groups from the respective non-NH_4_Cl-treated control groups.

### Differential scanning fluorimetry

The thermal stability (*T*_m_) of TcdB2 in the presence of soluble chondroitin sulfate or hyaluronic acid (51 kDa, Lifecore, Minnesota) was determined by performing thermal melt curves on a QuantStudio 5 Real-Time PCR system (Applied Biosystems) with the excitation–emission filter set to x1(470 ± 15)–m3(586 ± 10). These 20-µL reactions were performed in technical quadruplicate with 3 µg of TcdB and 50–200 μM of either chondroitin sulfate A, chondroitin sulfate C, or hyaluronic acid in HBSS ± 5 mM CaCl_2_, pH 7. 6.25X SYPRO Orange (Invitrogen, #S6653) was added to each sample, and the fluorescence emission was monitored as the temperature stepped from 25°C to 99°C at a rate of 0.05°C/s. The *T_m_* was calculated by taking the inverse of the first derivative of each reaction. The reactions were performed three independent times.

### Statistical analysis

Results were analyzed in the statistical software program Prism v10.0.0 using a two-way ANOVA followed by Šídák’s multiple comparisons test or a one-way ANOVA with Dunnett’s multiple comparisons test.

## Data Availability

Raw data sets for receptor binding, flow cytometry, cytotoxicity, and differential scanning fluorimetry measurements are available upon request.

## SUPPLEMENTAL MATERIAL

Supplemental figures msphere.00094-24-s0001.pdf Fig. S1 to S5.

ASM does not own the copyrights to Supplemental Material that may be linked to, or accessed through, an article. The authors have granted ASM a non-exclusive, world-wide license to publish the Supplemental Material files. Please contact the corresponding author directly for reuse.
