# Supplementary material for: CSPG4-dependent cytotoxicity for C. difficile TcdB is influenced by extracellular calcium and chondroitin sulfate
Source: mSphere. 2024 Mar 12;9(4):e00094-24. doi: 10.1128/msphere.00094-24 (PMC11036797; doi:10.1128/msphere.00094-24)
Supplement: Supplemental figures — Fig. S1 to S5. [file msphere.00094-24-s0001.pdf]

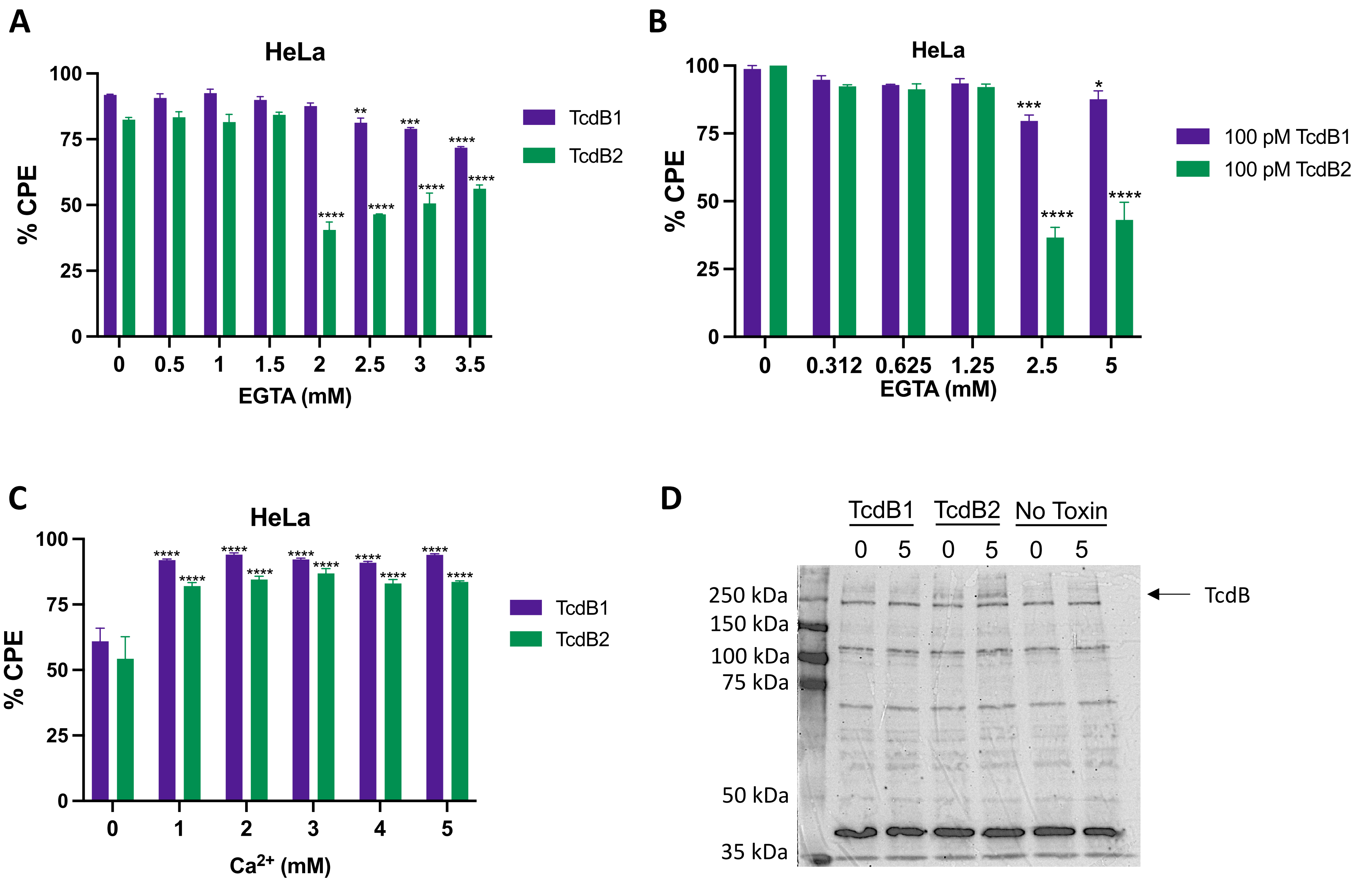

Supporting Figure 1. Cytotoxicity assays were used to quantify the % cytopathic effects (% CPE) in Hela cells pretreated with EGTA (*A*) following a 6 h treatment with 10 pM TcdB1 or TcdB2, (*B*) following a 3 h treatment with 100 pM TcdB1 or TcdB2. Cytotoxicity assays were used to quantify the %CPE in HeLa cells or (*C*) following a 6 h treatment with 10 pM TcdB1 or TcdB2 in HBSS ± 5 mM $CaCl_2$. (*D*) Immunoblot of TcdB from HeLa cell lysates treated with 200 pM TcdB1 or TcdB2 for 30 min in divalent cation free HBSS with or without the addition of 5 mM $CaCl_2$. Results are given as mean ± standard error of the mean from a representative experiment. Each experiment was repeated three independent times with similar results. Statistical significance for each experiment was calculated using a Two-Way ANOVA with Šídák's multiple comparison test. *, $P \leq 0.03$; **, $P \leq 0.002$; ***, $P \leq 0.0002$; ****, $P \leq 0.0001$.

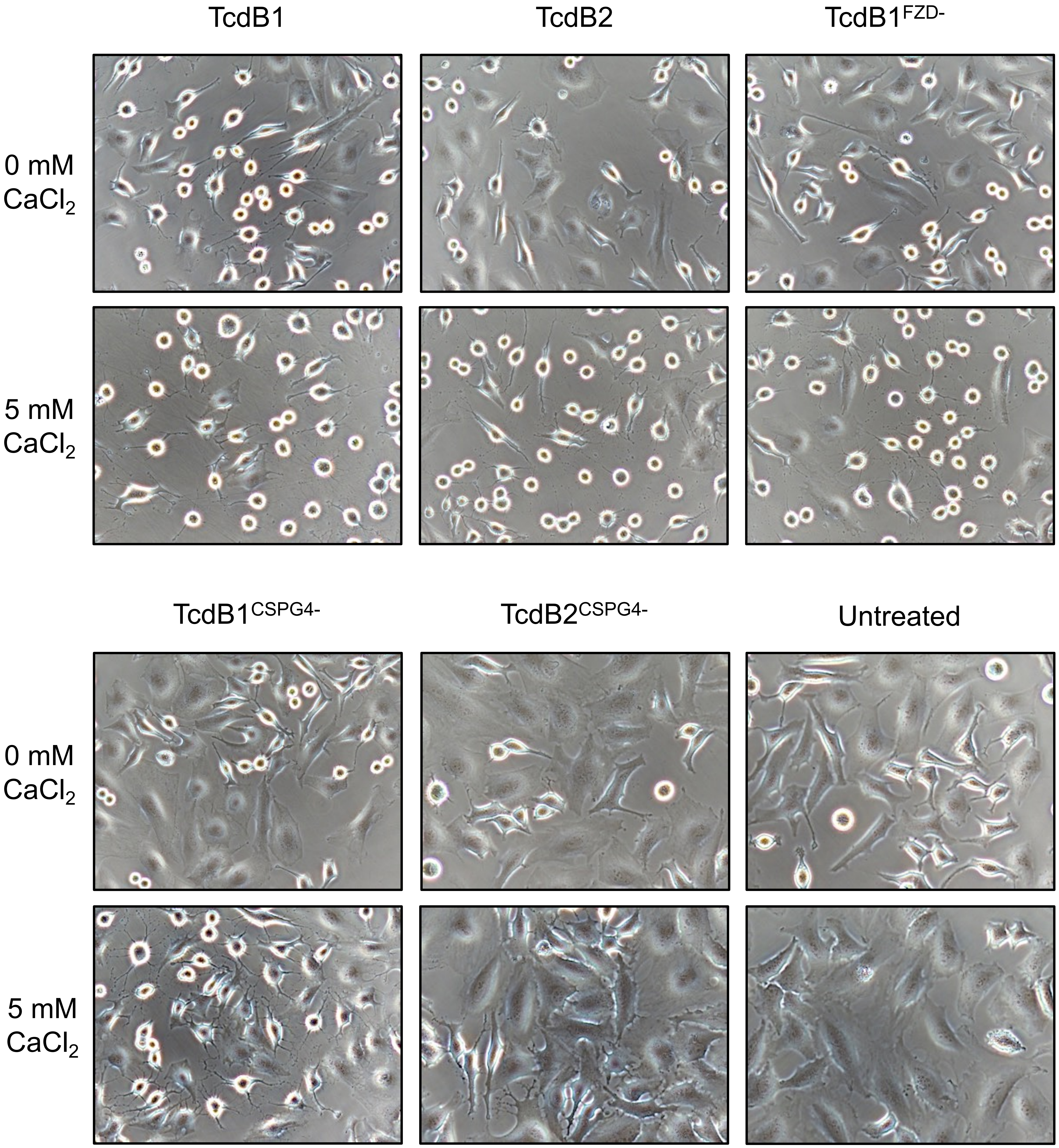

Supporting Figure 2. Representative microscopy images of HeLa cells treated with 10 pM TcdB1, TcdB2, TcdB1^{FZD-}, TcdB1^{CSPG4-}, or TcdB2^{CSPG4-} in HBSS ± 5 mM $CaCl_2$ after 3 h. Cells intoxicated by TcdB can be distinguished from non-intoxicated cells if the cell is > 95% rounded in appearance due to cytoskeletal collapse.

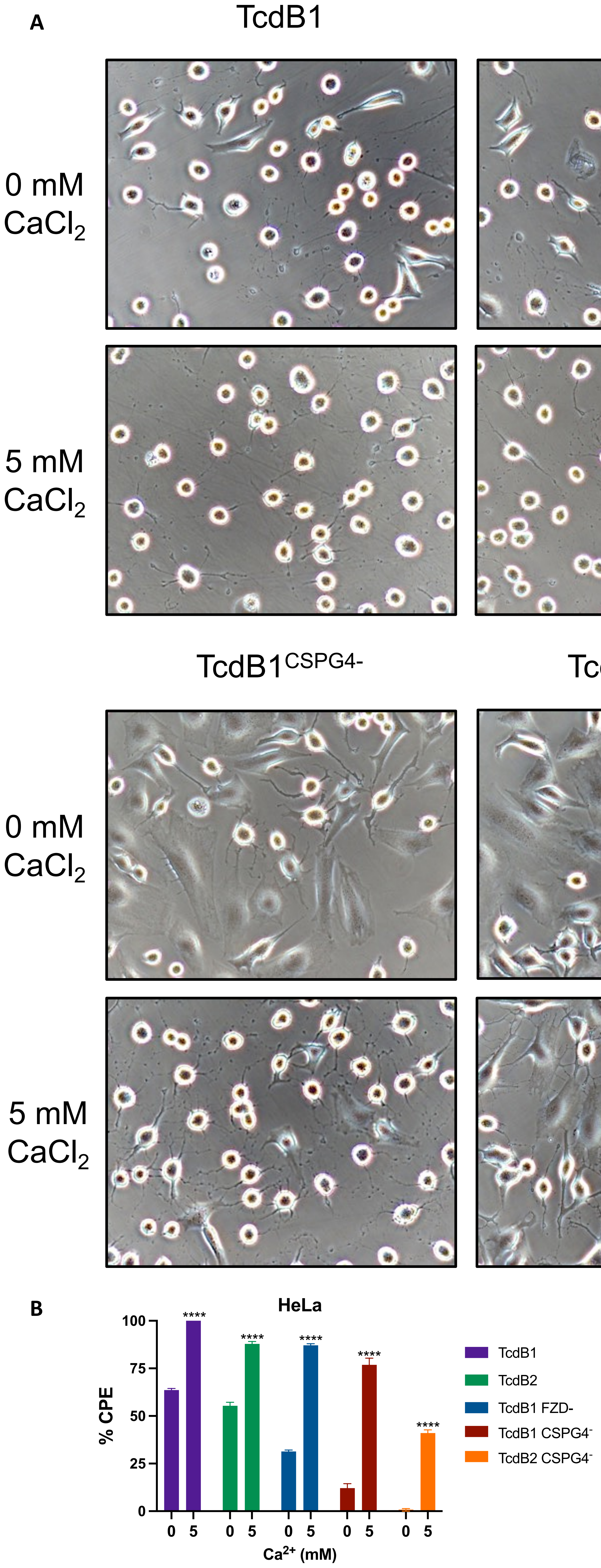
A
TcdB1
TcdB2
TcdB1^{FZD-}
0 mM CaCl$_2$
5 mM CaCl$_2$
TcdB1^{CSPG4-}
TcdB2^{CSPG4-}
Untreated
0 mM CaCl$_2$
5 mM CaCl$_2$
B
HeLa
% CPE
100
75
50
25
0
****
0 5 0 5 0 5 0 5 0 5
Ca^{2+} (mM)
TcdB1
TcdB2
TcdB1 FZD-
TcdB1 CSPG4$^-$
TcdB2 CSPG4$^-$

Supporting Figure 3. (*A*) Representative microscopy images of HeLa cells treated with 10 pM TcdB1, TcdB2, $TcdB1^{FZD-}$, $TcdB1^{CSPG4-}$, or $TcdB2^{CSPG4-}$ in HBSS ± 5 mM $CaCl_2$ after 6 h. (*B*) Quantification of the % cytopathic effects (% CPE) in HeLa cells (n=3) following a 6 h treatment with 10 pM TcdB1, TcdB2, $TcdB1^{FZD-}$, $TcdB1^{CSPG4-}$, or $TcdB2^{CSPG4-}$ in HBSS ± 5 mM $CaCl_2$. Cells intoxicated by TcdB can be distinguished from non-intoxicated cells if the cell is > 95% rounded in appearance due to cytoskeletal collapse. Each experiment was repeated three independent times with similar results. Statistical significance for each experiment was calculated using a Two-Way ANOVA with Šídák's multiple comparison test. ****, P ≤ 0.0001.

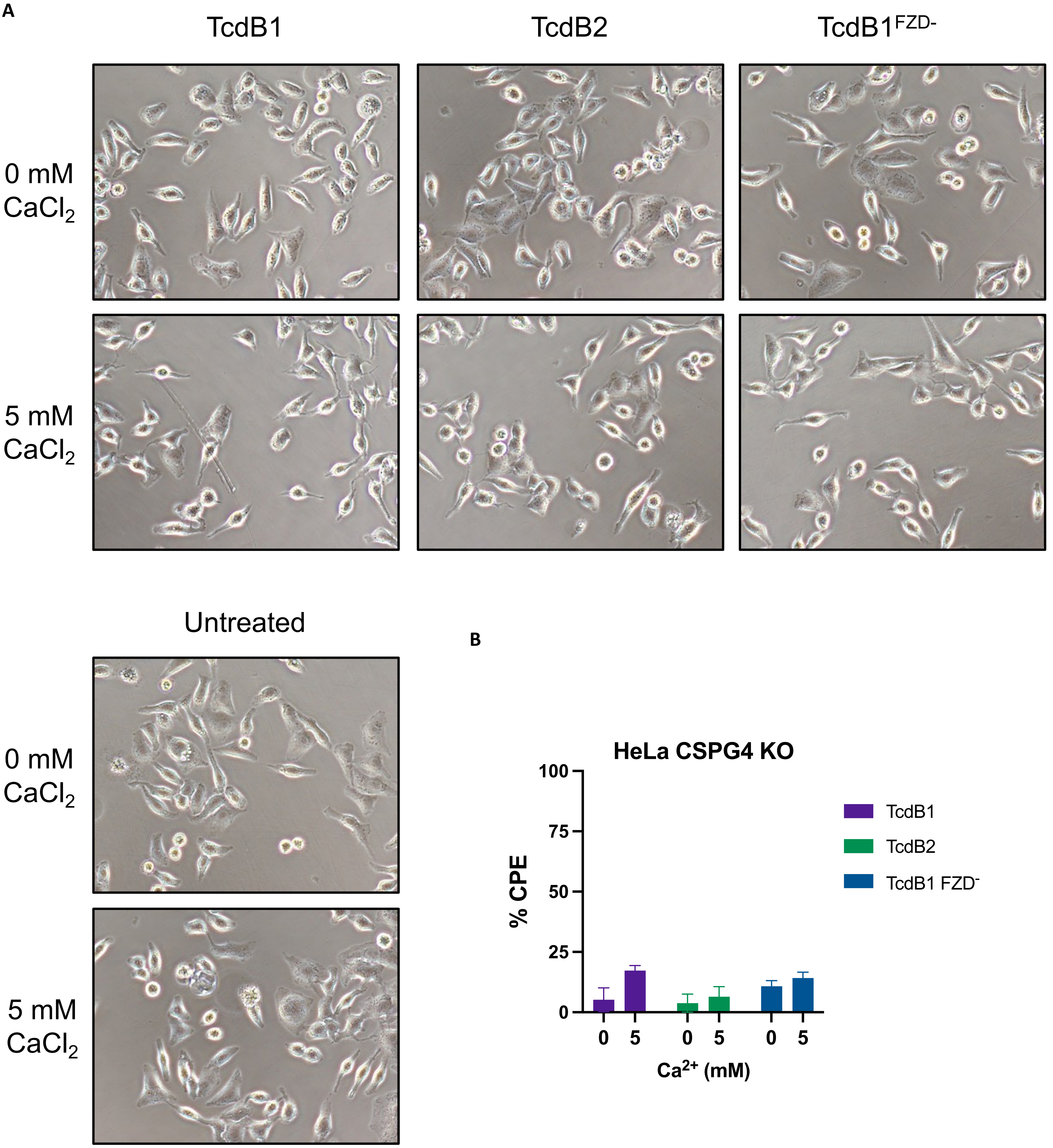

Supporting Figure 4. (*A*) Representative microscopy images of HeLa$^{CSPG4-/-}$ cells treated with 10 pM TcdB1, TcdB2 or TcdB1^{FZD-} in HBSS ± 5 mM $CaCl_2$ after 6 h. (*B*) Quantification of the % cytopathic effects (% CPE) in HeLa$^{CSPG4-/-}$ cells (n=3) following a 6 h treatment with 10 pM TcdB1, TcdB2, or TcdB1^{FZD-} in HBSS ± 5 mM $CaCl_2$. Cells intoxicated by TcdB can be distinguished from non-intoxicated cells if the cell is > 95% rounded in appearance due to cytoskeletal collapse. Each experiment was repeated three independent times with similar results. Statistical significance for each experiment was calculated using a Two-Way ANOVA with Šídák's multiple comparison test. There was no statistical significance between these sample groups.

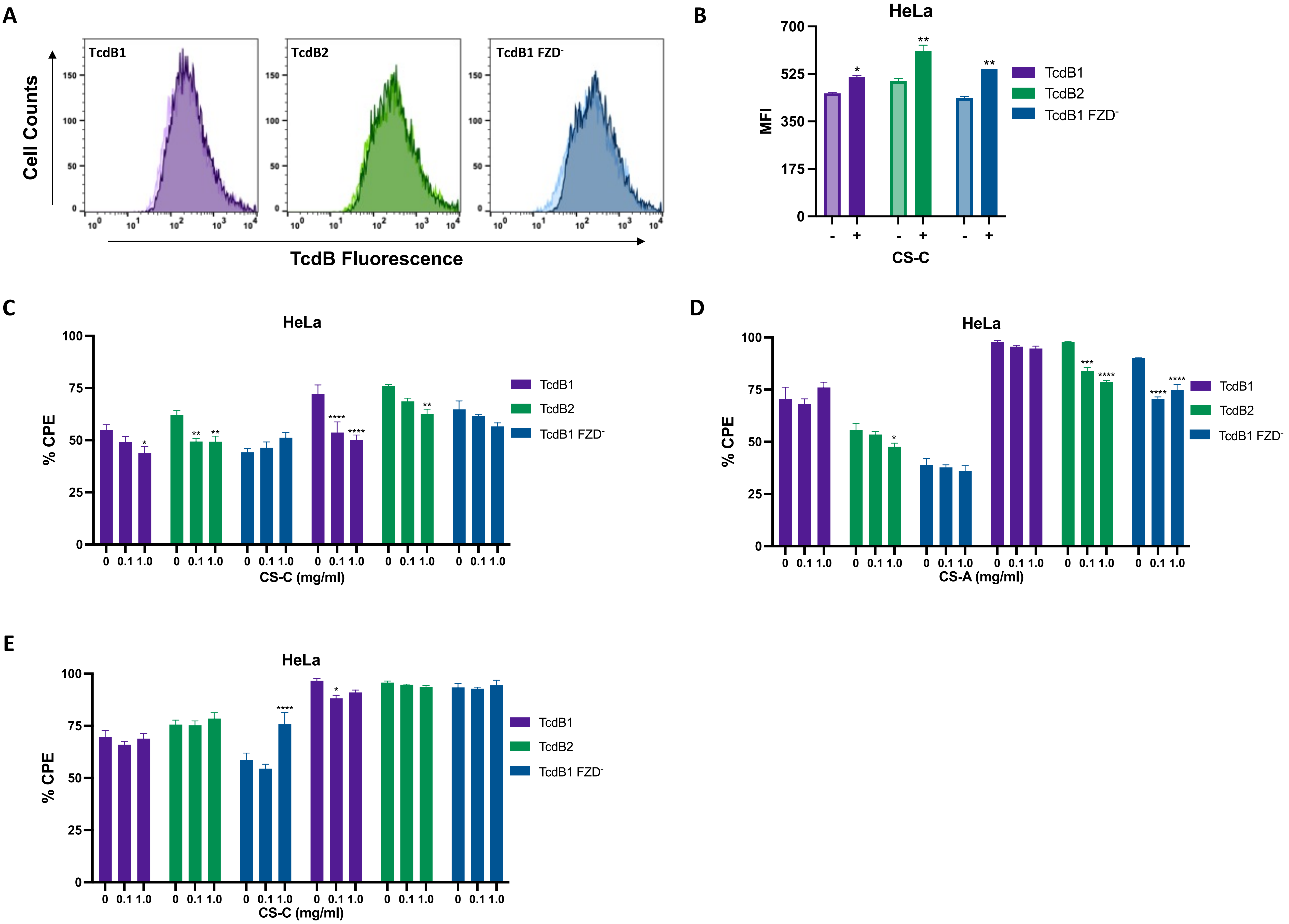

Supporting Figure 5. TcdB binding to HeLa cells in the presence of soluble chondroitin sulfate C was measured by flow cytometry. Cells were exposed to a mixture of 30 nM $TcdB1_{Alexafluor647}$, $TcdB2_{Alexafluor647}$, or $TcdB1^{FZD-}_{Alexafluor647}$ with 8.0 mg/ml CS-C in HBSS + 5 mM $CaCl_2$ for 10 min at 37℃ followed by 20 min on ice before a series of washes and assessment by flow cytometry. (*A*) Histogram comparing the fluorescent signal from HeLa cells exposed to $TcdB1_{Alexafluor647}$, $TcdB2_{Alexafluor647}$, or $TcdB1^{FZD-}_{Alexafluor647}$ in the presence (dark color shade) or absence (light color shade) of chondroitin sulfate. (*B*) Mean Fluorescent Intensity (MFI) of flow cytometry data from a representative experiment. Results are given as mean ± standard error of the mean from a representative experiment given as a mean ± standard error of the mean. Each experiment was repeated two independent times with similar results. Cytotoxicity assays were used to quantify the % cytopathic effects (% CPE) in Hela cells following (*C*) a 2 h treatment of 10 pM TcdB1, TcdB2, or $TcdB1^{FZD-}$ mixed with soluble CS-C in the presence or absence of 5 mM $CaCl_2$, or a 4 h treatment of 10 pM TcdB1, TcdB2 or $TcdB1^{FZD-}$ mixed with soluble CS-A (*D*) or CS-C (*E*) in the presence or absence of 5 mM $CaCl_2$. Results are given as mean ± standard error of the mean from a representative experiment. Each experiment was repeated three independent times with similar results. Statistical significance for each experiment was calculated using a Two-Way ANOVA with Šídák's multiple comparison test. *, $P \le 0.03$; ***, $P \le 0.0002$; ****, $P \le 0.0001$.
